# Vitamin D and sudden sensorineural hearing loss: emerging associations, mechanistic insights, and prospects for precision medicine — a review

**DOI:** 10.3389/fnut.2026.1844126

**Published:** 2026-05-25

**Authors:** Ying Wang, Zhen Pan, Xingjia Hu

**Affiliations:** Department of Otolaryngology Head and Neck Surgery, Changde Hospital, Xiangya School of Medicine, Central South University (The First People's Hospital of Changde City), Changde, China

**Keywords:** candidate prognostic marker, etiology, hearing loss, precision medicine, review, sudden sensorineural hearing loss, vitamin D

## Abstract

Sudden sensorineural hearing loss (SSNHL) is defined as a rapid hearing decline of ≥30 dB across at least three contiguous frequencies within 72 h, with a multifactorial etiology encompassing vascular, viral, autoimmune, and metabolic mechanisms. Beyond its classical role in calcium and phosphorus homeostasis, vitamin D exerts broad biological effects, including immunomodulation, antioxidant defense, endothelial protection, and neuroprotection, which may collectively preserve inner ear function. Multiple observational studies have demonstrated that serum 25-hydroxyvitamin D levels are significantly lower in SSNHL patients than in healthy controls, with deficiency associated with greater hearing impairment, poorer treatment response, and higher recurrence risk. However, it must be emphasized that no standardized serum threshold, population-specific validation, or universally accepted predictive model currently exists; therefore, serum 25(OH)D should be regarded as a candidate—rather than an established—biomarker for SSNHL risk stratification. The causal relationship between vitamin D status and SSNHL remains unestablished, with a Mendelian randomization study finding no significant causal link, and inconsistencies across intervention trials highlighting uncertainties regarding optimal dosage and treatment timing. This review synthesizes current evidence on the mechanisms, clinical associations, and exploratory therapeutic potential of vitamin D in SSNHL, and proposes that future prospective, multicenter studies integrating vitamin D metabolomics and receptor gene polymorphism analyses are essential to identify patient subgroups most likely to benefit, ultimately advancing vitamin D as a candidate biomarker and a potential adjunctive therapeutic consideration in the precision management of SSNHL.

## 1 Introduction

Sudden sensorineural hearing loss (SSNHL) is defined as a rapid hearing loss of ≥30 dB across at least three contiguous frequencies within 72 h, with an annual incidence of approximately 5–27 per 100,000 individuals. Although it can occur at any age, it is most prevalent among those aged 40–60 years. While some patients experience spontaneous recovery, a significant proportion develops permanent hearing impairment, severely affecting quality of life and social function. The etiology of SSNHL is complex, involving viral infection, vascular embolism, autoimmune reactions, and inner ear metabolic disorders. Currently, no unified diagnostic or therapeutic standard exists, and conventional treatments such as glucocorticoids demonstrate considerable individual variability in efficacy. Early identification of high-risk populations, prediction of treatment response, and formulation of individualized intervention strategies remain major challenges in otolaryngology. Recently, growing research has focused on the potential role of nutritional and metabolic factors in SSNHL pathogenesis, with vitamin D deficiency emerging as a key research focus due to its high prevalence and modifiability (1–3).

Vitamin D participates not only in calcium and phosphorus metabolism and skeletal homeostasis, but also exerts broad non-classical biological effects, including immune modulation, antioxidant stress resistance, endothelial protection, and neuroprotection. The molecular basis of these pleiotropic effects is now well characterized. Vitamin D operates through two principal signaling modes: (i) genomic signaling, wherein the active form 1,25-dihydroxyvitamin D3 (calcitriol) binds to the nuclear vitamin D receptor (VDR), which subsequently heterodimerizes with the retinoid X receptor (RXR) to regulate vitamin D response elements (VDREs) in target gene promoters, modulating the transcription of hundreds of genes involved in immune defense, oxidative stress, and cellular homeostasis; and (ii) non-genomic signaling, wherein membrane-bound VDR or alternative receptors initiate rapid second-messenger cascades—including activation of protein kinase C (PKC), phospholipase C (PLC), and mitogen-activated protein kinase (MAPK) pathways—within seconds to minutes (4). These dual pathways collectively explain vitamin D’s broad immunomodulatory, anti-inflammatory, and cytoprotective capacities. The VDR is widely distributed throughout systemic tissues—including the inner ear—enabling calcitriol to regulate the expression of genes critical for cochlear homeostasis. Animal studies have demonstrated that VDR deletion leads to degenerative changes in inner ear ganglia, hair cells, and otoliths, suggesting a critical role of vitamin D in maintaining the structural and functional integrity of the inner ear (5). Furthermore, in a mouse model of noise-induced hearing loss, calcitriol significantly attenuated cochlear hair cell apoptosis by modulating the ATF3/DUSP1 signaling pathway to suppress oxidative stress and DNA damage (6). These findings provide the molecular basis for a protective role of vitamin D in sensorineural hearing disorders.

Multiple clinical studies have revealed associations between vitamin D deficiency and various sensorineural disorders. Case–control studies showed that serum 25-hydroxyvitamin D levels in SSNHL patients were significantly lower than in healthy controls (19.28 ± 9.56 vs. 25.71 ± 11.21 ng/mL, *p* < 0.001, or 26.55 ± 14.44 vs. 33.51 ± 14.21 ng/mL, *p* = 0.017), with vitamin D deficiency being more prevalent among patients (1, 3). Cross-sectional analyses further confirmed that vitamin D deficiency (<20 ng/mL) was significantly associated with bilateral low-frequency and speech-frequency sensorineural hearing loss in adults over 50 years (adjusted OR = 1.45–1.60) (7), with similar associations observed in elderly populations (OR = 1.96–2.02) (8). Additionally, vitamin D deficiency has been independently associated with tinnitus, Meniere’s disease, and sensorineural hearing loss in children (9–13). Notably, a prospective cohort study found that vitamin D deficiency was associated with the onset and recurrence of SSNHL, with the strongest association in individuals under 30 years (14). Importantly, it must be acknowledged that the majority of these associations derive from observational studies subject to potential confounding by sun exposure, lifestyle, and comorbidities. Although a Mendelian randomization study found no causal relationship between genetically predicted vitamin D levels and SSNHL (15), most observational and interventional studies support a close association between vitamin D status and inner ear function, suggesting its potential as a candidate modifiable risk factor or prognostic biomarker—albeit one requiring causal validation—in the prevention and treatment of sensorineural diseases.

## 2 Molecular mechanisms by which vitamin D affects inner ear function

**Figure 1:**
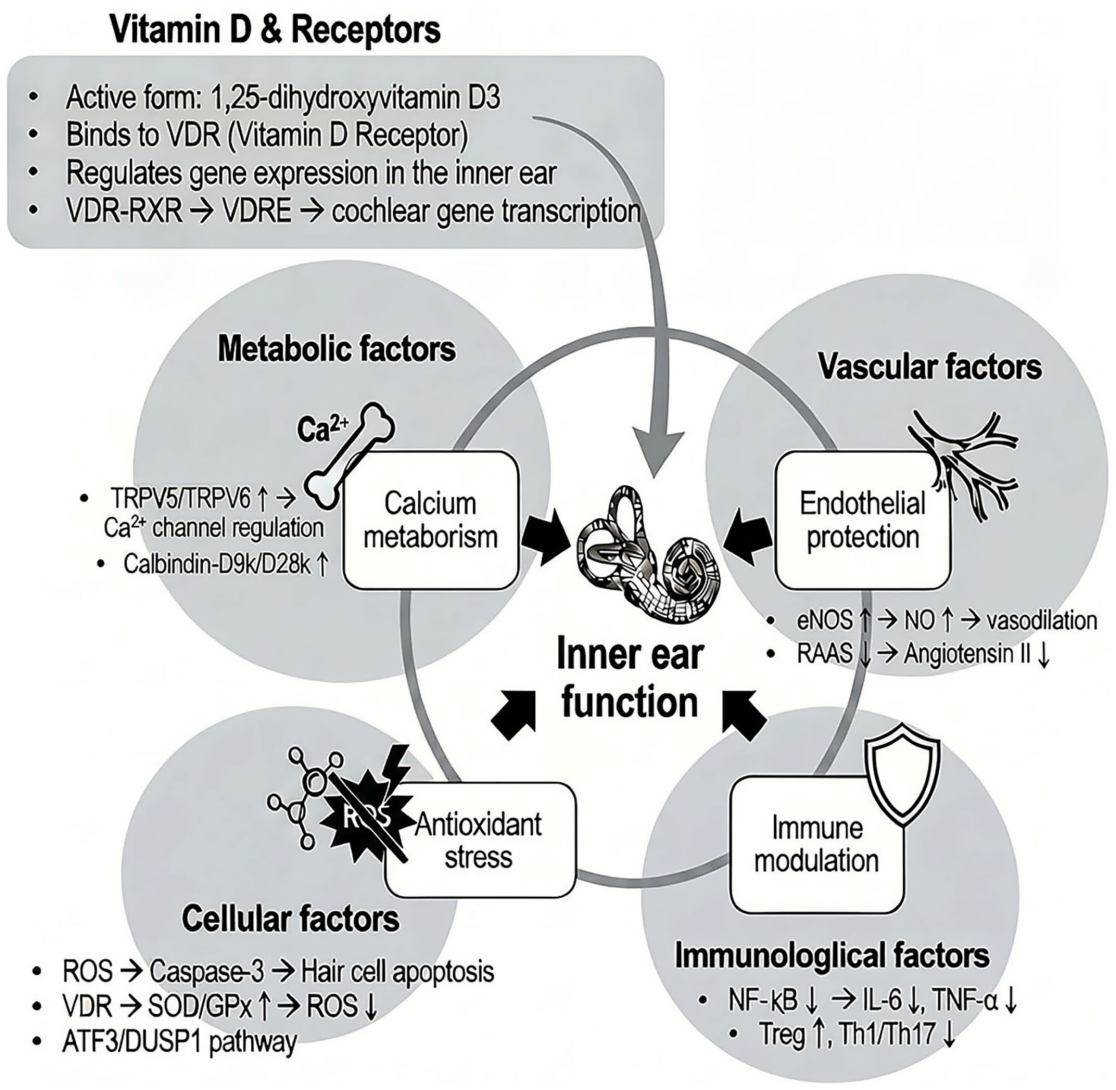
Summary of the molecular mechanisms linking vitamin D to inner ear function.

The molecular mechanisms linking vitamin D to inner ear function are summarized in Figure 1. Rather than operating through independent pathways, these mechanisms converge within cochlear pathophysiology: calcium dysregulation sensitizes hair cells to oxidative insults; oxidative stress amplifies inflammatory cascades via NF-κB activation; neuroinflammation impairs microvascular tone; and endothelial dysfunction exacerbates tissue hypoxia and hair cell death. Vitamin D, acting through VDR-mediated transcriptional networks, simultaneously attenuates each of these interconnected processes, providing a unified framework for its cochlear-protective role (4).

### 2.1 Calcium metabolism regulation and cochlear hair cell homeostasis

As a key regulator of calcium and phosphorus metabolism, calcitriol binds to VDR to regulate the expression of multiple calcium channels and transporters, thereby maintaining intracellular and extracellular calcium ion homeostasis. Specifically, VDR activation upregulates the epithelial calcium channels TRPV5 and TRPV6, as well as the calcium-binding proteins calbindin-D9k and calbindin-D28k, which are essential for transcellular calcium transport (16). Dysregulation of these channels disrupts the precise endolymphatic calcium gradient ([Ca^2+^] ~ 20–30 μM) that is critical for mechanotransduction in cochlear hair cells (17). In the inner ear, cochlear hair cells are highly sensitive to calcium signals, and their normal function depends on precise calcium concentration gradients. Animal studies have demonstrated that VDR deletion leads to degenerative changes in inner ear ganglia, hair cells, and otolith structures, indicating an important role of vitamin D in maintaining cochlear structural integrity (5). Furthermore, a study of women with vitamin D deficiency found significantly lower TEOAE responses at 3 and 4 kHz and DPOAE responses at 1, 2, and 6 kHz compared to controls, suggesting that vitamin D deficiency is closely associated with outer hair cell dysfunction (18). These findings collectively support the involvement of vitamin D in cochlear hair cell homeostasis through calcium metabolism regulation. Importantly, the biomarker implications of measuring serum 25(OH)D alone are limited by the fact that tissue-level calcium homeostasis is also regulated by local CYP27B1 enzymatic activity and VDR sensitivity, which may vary independently of circulating 25(OH)D concentrations (16).

### 2.2 Immunomodulatory function in autoimmune hearing loss

Recent studies have confirmed that the inner ear is not a strictly “immune-privileged” organ; it harbors resident macrophages, T lymphocytes, and dendritic cells, all regulated by the blood-labyrinth barrier (19). Immune dysregulation can trigger autoimmune inner ear disease, leading to sensorineural hearing loss. Vitamin D exerts significant immunomodulatory effects by inhibiting pro-inflammatory cytokines (such as IL-6 and TNF-*α*) and promoting anti-inflammatory factor (such as IL-10) release to regulate local immune responses (20). At the molecular level, VDR activation suppresses NF-κB signaling—a master regulator of pro-inflammatory gene transcription—thereby reducing the production of downstream inflammatory mediators including IL-1β, IL-6, TNF-*α*, and COX-2. Simultaneously, calcitriol promotes the differentiation of regulatory T cells (Tregs) and inhibits Th1 and Th17 cell polarization, shifting the immune balance toward tolerance (4). In a guinea pig otitis media model, vitamin D deficiency significantly exacerbated inflammatory pathological changes, manifesting as thickened middle ear mucosa and elevated pro-inflammatory factors (20). Clinical observations suggest that vitamin D may reduce the need for intratympanic gentamicin treatment in Meniere’s disease by suppressing post-viral autoimmune responses (21). Additionally, a case of progressive sensorineural hearing loss in a patient with LGI-1 antibody-associated autoimmune encephalitis further supports the role of autoimmune mechanisms in peripheral auditory pathway damage (22). Overall, vitamin D may exert protective effects in autoimmune hearing loss by modulating both innate and adaptive immune responses.

### 2.3 Vascular endothelial protection and cochlear microcirculation improvement

The cochlea is highly dependent on stable microcirculation for oxygen supply, and any factor causing microvascular dysfunction can induce tissue hypoxia and hearing impairment. Hypertension and hyperfibrinogenemia have been shown to impair cochlear blood flow, leading to elevated hearing thresholds (23, 24). Vitamin D can improve microcirculation by stabilizing endothelial cell function, inhibiting inflammatory factor release, and regulating vascular tone. Mechanistically, calcitriol suppresses the renin-angiotensin-aldosterone system (RAAS) by downregulating renin gene transcription through VDR-mediated promoter binding, thereby reducing angiotensin II-induced vasoconstriction and endothelial oxidative stress (25). Additionally, VDR activation enhances endothelial nitric oxide synthase (eNOS) expression, increasing nitric oxide (NO) bioavailability and promoting vasodilation within the stria vascularis (26). Animal experiments have demonstrated that tumor necrosis factor (TNF) can significantly reduce cochlear microcirculatory blood flow, and targeted intervention in this pathway can reverse blood flow impairment (27, 28). Notably, VDR can upregulate brain natriuretic peptide (BNP) expression in rat spiral ganglion neurons and promote neuronal survival and axonal elongation through the cGMP-PKG signaling pathway, indirectly supporting cochlear blood flow regulation and neural regeneration (29). Furthermore, SSNHL patients commonly exhibit systemic microcirculatory dysfunction and reduced cerebral perfusion, correlated with oxidative stress marker levels (30), suggesting that the endothelial protective effect of vitamin D may play a key regulatory role in this process (5).

### 2.4 Neuroprotective effects of antioxidant stress resistance

Oxidative stress is an important pathological mechanism underlying noise-induced, age-related, and sudden sensorineural hearing loss. Noise exposure can induce elevated pro-inflammatory cytokines (such as IL-1β, IL-6, and TNF-*α*) in the cochlea, causing damage to the stria vascularis and spiral ligament vascular structures, accompanied by downregulation of vasodilatory genes and upregulation of vasoconstrictive genes (31). At the cellular level, excessive reactive oxygen species (ROS) trigger mitochondrial dysfunction, releasing cytochrome c and activating the intrinsic apoptotic pathway (caspase-9/caspase-3 cascade) in cochlear hair cells (32). Vitamin D attenuates this cascade by upregulating endogenous antioxidant enzymes—including superoxide dismutase (SOD) and glutathione peroxidase (GPx)—and by modulating the ATF3/DUSP1 signaling pathway, which suppresses ROS-mediated DNA damage and apoptosis (6). NF-κB-driven inflammatory amplification of oxidative injury is also attenuated by VDR signaling, linking the antioxidant and anti-inflammatory pathways described above. In a mouse model of noise-induced hearing loss, calcitriol effectively suppressed oxidative stress and apoptosis in cochlear nerve fibers and spiral neurons through the ATF3/DUSP1 signaling pathway, thereby reducing hearing threshold shift (6). Clinical studies have also found that serum vitamin D levels in SSNHL patients are significantly lower than in healthy controls, with higher non-response rates among vitamin D-deficient patients, suggesting that its antioxidant effects may influence treatment outcomes (2, 3). Moreover, vitamin D deficiency is independently associated with hearing loss in diabetic patients—a population commonly exhibiting enhanced systemic oxidative stress (33)—further supporting the neuroprotective role of vitamin D through mitigation of oxidative damage.

## 3 Clinical characteristics of SSNHL and association with vitamin D

Clinical studies on vitamin D and sudden sensorineural hearing loss are summarized in Table 1. It should be noted that the included studies vary considerably in design quality, sample size, population characteristics, and vitamin D deficiency definitions, which limits direct cross-study comparisons. Case–control studies can identify associations but are prone to selection and confounding biases; prospective cohorts provide temporal information but may not establish causality; and the available randomized controlled trials, while providing the highest level of interventional evidence, are small in sample size and heterogeneous in dosing protocols.

**Table 1:** Summary of clinical studies on vitamin D and SSNHL.

Study design	Participants (*n*)	Age (years, mean ± SD)	Sex (M/F)	Vitamin D status at baseline [25(OH)D, mean ± SD, ng/mL]	Vitamin D deficiency definition and rate	Intervention (vitamin D dose and duration)	Key findings	Reference
Case–control study	SSNHL: 310/HC: 154	SSNHL: 44.03 ± 12.28/HC: 45.58 ± 9.34	233/231	M: SSNHL:41.1 ± 12.97/HC:50.36 ± 9.86 F: SSNHL:35.90 ± 10.61/HC: 44.68 ± 10.04	<12 ng/mL; SSNHL: 16.3% (M), 29.9% (F)/HC: 0%	None	Vitamin D deficiency observed exclusively in SSNHL patients, not in controls	(1)
Case–control study	SSNHL: 34/HC: 34	SSNHL: 50.26 ± 15.89/HC: 48.12 ± 15.75	26/42	SSNHL: 19.28 ± 9.56/HC: 25.71 ± 11.21	<12 ng/mL; SSNHL: 26.5%/HC: 8.8%	None	All vitamin D-sufficient patients achieved complete recovery; 87.5% of deficient patients had no response to treatment.	(2)
Case–control study	SSNHL: 50/HC: 50	SSNHL: 50.66 ± 16.43/HC: 44.22 ± 15.11	55/45	SSNHL: 26.55 ± 14.44/HC: 33.51 ± 14.21	<20 ng/mL; SSNHL: 70%/HC: 44%	None	Vitamin D deficiency significantly associated with increased SSNHL risk	(3)
Prospective cohort study	SSNHL: 80/HC: 60	SSNHL: 41.2 ± 11.9/HC: 44.0 ± 14.8	76/64	SSNHL: 18.4 ± 4.1/HC: 32.6 ± 11.0	<20 ng/mL; SSNHL: 38.8%/HC: 10.0%	oral vitamin D2 (800IU/day) and D3 (250 IU/day)for 3 months after diagnosis	Vitamin D deficiency is a modifiable risk factor for SSNHL onset and recurrence, especially in patients <30 years; short-term supplementation did not significantly improve recovery (75.7% vs. 68.2%)	(14)
Randomized controlled trial	Intervention: 50 (VDD-SSNHL)/control: 51 (VDD-SSNHL)	Intervention: 45.58 ± 14.62/control: 42.67 ± 15.34	43/58	Intervention: 45.59 ± 12.06/ Control: 37.91 ± 12.31	<30 ng/mL; 100% (inclusion criterion)	Vitamin D3 1,500–2000 IU/day orally × 10 days	Recovery rate: 82.0% (10-day) and 76.0% (3-month) vs. 52.9% and 47.1% in controls; PTA improvement: 29.3 vs. 14.2 dB HL (10-day), 25.1 vs. 12.5 dB HL (3-month); THI reduction: 27.5 vs. 13.9	(39)
Randomized controlled trial	Intervention: 20 (VDD-SSNHL)/control: 20(VDD-SSNHL)	Intervention: 45.9 ± 17.5/control: 37 ± 12.5	22/18	Intervention: 13.24 ± 3.99/control: 17.22 ± 6.13	<20 ng/mL; 100% (inclusion criterion)	Vitamin D3 50,000 IU/week orally × 3 weeks	Significant improvement at 2000 Hz and 4,000 Hz; no abnormal changes in serum calcium or phosphorus	(40)

HC, healthy controls; VDD, vitamin D deficiency; VDD-SSNHL, SSNHL patients with vitamin D deficiency; M, male; F, female; NR, not reported; PTA, pure-tone average; THI, tinnitus handicap inventory; RCT, randomized controlled trial; 25(OH)D, 25-hydroxyvitamin D. For RCT studies (31, 32), both the intervention and control groups consisted exclusively of SSNHL patients with confirmed vitamin D deficiency; healthy controls were not included. All participants in these trials received standard SSNHL therapy, with the intervention group additionally receiving vitamin D supplementation.

### 3.1 Pathophysiological features of idiopathic SSNHL

Idiopathic SSNHL is an unexplained sensorineural hearing decline occurring within 72 h, typically unilateral, with hearing threshold reduction of ≥30 dB HL in at least two adjacent frequencies. Its underlying pathological mechanisms are complex and may involve inner ear microcirculatory disorders, viral infection, autoimmune reactions, endolymphatic hydrops, and oxidative stress (5). Recently, increasing research has begun to focus on the role of systemic metabolic and endocrine factors in SSNHL pathogenesis, with vitamin D gaining attention for its broad biological functions. Clinical observations have found that patients often present with various systemic metabolic abnormalities, including vitamin D deficiency, suggesting its potential involvement in disease onset or progression (1).

### 3.2 Clinical phenotype analysis of patients with vitamin D deficiency

Multiple case–control studies consistently show that serum 25-hydroxyvitamin D levels in SSNHL patients are significantly lower than in normal-hearing populations. For example, a study of 310 SSNHL patients and 154 controls found that the mean vitamin D level in the patient group was significantly lower, with vitamin D deficiency seen only in the patient group (1). Another study of 50 patients showed that the mean vitamin D level in the SSNHL group was 26.55 ± 14.44 ng/mL, significantly lower than the control group’s 33.51 ± 14.21 ng/mL (*p* = 0.017), with a deficiency rate of up to 70% compared to only 44% in controls (3). Additionally, vitamin D-deficient SSNHL patients not only experienced more severe hearing loss but also showed poorer treatment responses: all vitamin D-sufficient patients achieved complete hearing recovery, while 87.5% of deficient patients had incomplete recovery (2). Notably, in younger patients (<30 years), the vitamin D deficiency rate can reach 60%, and vitamin D levels in recurrent patients were significantly lower than in first-episode patients (18.4 ± 4.1 vs. 24.9 ± 9.7 ng/mL) (14). However, these associations must be interpreted cautiously. Vitamin D status is confounded by multiple factors—including geographic latitude and seasonal sunlight exposure, dietary habits, body mass index, chronic systemic diseases (e.g., diabetes, hypertension, autoimmune conditions), and medication use—all of which may independently influence both vitamin D levels and SSNHL outcomes. The observed deficiency in SSNHL patients may therefore reflect general metabolic vulnerability rather than a specific causal pathway. These data collectively suggest that vitamin D deficiency may constitute a specific clinical phenotype of SSNHL, characterized by more severe hearing impairment, higher recurrence risk, and poorer treatment response—though this phenotypic classification requires prospective validation.

### 3.3 Association between vitamin D levels and prognosis in elderly patients

In elderly populations, the association between vitamin D deficiency and hearing loss is particularly prominent. Studies based on the NHANES database showed that in adults ≥70 years old, serum 25-hydroxyvitamin D levels below 20 ng/mL were significantly associated with low-frequency hearing loss (OR: 2.02; 95% CI: 1.28, 3.19) and speech-frequency hearing loss (OR: 1.96; 95% CI: 1.12, 3.44) (8). Another cross-sectional analysis found that in adults ≥50 years old, vitamin D deficiency (<20 ng/mL) was closely associated with bilateral low-frequency hearing loss (adjusted OR = 1.45, 95% CI 1.12–1.89) and bilateral sensorineural hearing loss (adjusted OR = 1.60, 95% CI 1.13–2.26) (7). These results suggest that elderly SSNHL patients with concurrent vitamin D deficiency may face more severe inner ear functional damage. Although long-term follow-up data specifically examining vitamin D intervention in elderly SSNHL patients are lacking, available evidence suggests that maintaining adequate vitamin D levels may have potential—though unproven—value for delaying age-related hearing deterioration and improving SSNHL prognosis (14). Therefore, assessing and correcting vitamin D status may serve as an important component of optimizing treatment strategies in elderly SSNHL management, pending confirmation from prospective interventional trials.

## 4 Progress in diagnostic biomarker research

### 4.1 Standardization of serum vitamin D level detection

Serum 25-hydroxyvitamin D [25(OH)D] has been widely adopted as the gold standard for assessing vitamin D status in SSNHL-related research. Multiple case–control studies consistently show that serum 25(OH)D levels in SSNHL patients are significantly lower than in healthy controls: one study reported mean levels of 19.28 ± 9.56 ng/mL in SSNHL patients vs. 25.71 ± 11.21 ng/mL in controls (*p* < 0.001) (2); another found 26.55 ± 14.44 vs. 33.51 ± 14.21 ng/mL (*p* = 0.017) in SSNHL vs. controls (3). Furthermore, the proportion of SSNHL patients with vitamin D insufficiency (<30 ng/mL) was as high as 70%, significantly higher than the 44% in controls (3), and some studies observed vitamin D deficiency exclusively in SSNHL patient populations (1).

Critically, serum 25(OH)D as a standalone biomarker has important limitations. First, 25(OH)D reflects hepatic hydroxylation capacity but not the renal conversion to the biologically active calcitriol [1,25(OH)₂D₃], which depends on CYP27B1 enzyme activity and is tightly regulated by PTH, FGF-23, and calcium feedback loops (34). Second, tissue-level VDR sensitivity varies substantially across individuals and is modulated by VDR gene polymorphisms (e.g., FokI, BsmI, TaqI, ApaI), meaning that equivalent serum 25(OH)D concentrations may produce markedly different biological responses (35). Third, chronic inflammation—common in SSNHL—can suppress VDR expression and blunt calcitriol’s biological effects independently of circulating 25(OH)D levels. These complexities underscore that serum 25(OH)D alone may not fully capture functional vitamin D biological activity in the cochlear microenvironment. Although these studies have not fully unified detection methods and reference ranges, all use serum 25(OH)D concentration as the core indicator, suggesting its value in SSNHL risk stratification and potential screening. However, standardized protocols for vitamin D testing in SSNHL populations—including sampling timing, storage conditions, and platform consistency—are currently lacking, limiting its promotion as a routine clinical biomarker (36).

### 4.2 Combined assessment of endothelial function markers and vitamin D status

Endothelial dysfunction is considered one of the important pathological mechanisms of SSNHL, while vitamin D has potential vascular endothelial protective effects. Although existing literature has not directly performed combined modeling analyses of vitamin D and endothelial markers, some studies indirectly support their association in SSNHL. For example, fibrinogen—an indicator of coagulation status and endothelial damage—has been shown to be closely associated with SSNHL prognosis, with high fibrinogen levels indicating poorer hearing recovery (37). Simultaneously, vitamin D deficiency may impair microcirculation by affecting endothelial nitric oxide synthase activity and promoting inflammatory responses, thereby affecting cochlear blood supply (36). Future research should perform combined assessment of serum 25(OH)D levels with endothelial function markers (such as von Willebrand factor, endothelin-1, and fibrinogen) to construct more comprehensive SSNHL risk prediction models. Currently, such integrated biomarker strategies have not been established in clinical practice, though their theoretical basis has begun to emerge in multi-mechanism models of sensorineural hearing loss (36).

### 4.3 Auxiliary diagnostic value of inflammatory cytokine profiles

Inflammatory responses play an important role in SSNHL pathogenesis, with multiple studies confirming a state of systemic inflammatory activation in patients. Systematic reviews have noted that SSNHL patients commonly exhibit elevated inflammatory markers, suggesting possible autoimmune or chronic low-grade inflammatory processes (38). Conventional inflammatory indicators such as complete blood counts and C-reactive protein, while non-specific, are recommended for preliminary assessment due to their low cost and accessibility (38). Some novel inflammation-related molecules such as heat shock protein-70 and anti-endothelial cell antibodies have been proposed as potential biomarkers but require verification through prospective studies (38). Notably, vitamin D itself has significant immunomodulatory functions, capable of inhibiting pro-inflammatory cytokine (such as IL-6, TNF-*α*) production and promoting regulatory T cell differentiation (36). Although direct correlational data between vitamin D levels and specific inflammatory factors in SSNHL have not been reported, studies have linked vitamin D deficiency to poor treatment responses—for example, all vitamin D-sufficient patients achieved complete hearing recovery while 87.5% of deficient patients had incomplete recovery (2)—indirectly suggesting that inflammatory regulation may mediate the protective effects of vitamin D. Therefore, incorporating serum 25(OH)D levels into integrated analysis frameworks of inflammatory cytokine profiles (such as IL-6, CRP, and fibrinogen) is expected to improve early identification rates and prognostic accuracy for SSNHL, though direct evidence supporting the clinical utility of this combined strategy is currently lacking (38).

## 5 Evidence-based medicine for treatment strategies

### 5.1 Clinical efficacy evaluation of vitamin D supplementation

Recent clinical studies have investigated the role of vitamin D supplementation in SSNHL treatment. A prospective randomized controlled trial enrolled vitamin D-deficient SSNHL patients and administered daily 1,500–2000 IU vitamin D3 combined with conventional treatment (methylprednisolone plus *ginkgo biloba* extract), showing short-term (10-day) and long-term (3-month) hearing recovery rates of 82.0 and 76.0%, respectively, significantly higher than the 52.9 and 47.1% in the conventional treatment-only group. The mean pure-tone threshold improvement was also greater (10 days, 29.3 dB HL vs. 14.2 dB HL, 3 months: 25.1 dB HL vs. 12.5 dB HL), and the tinnitus handicap inventory score reduction was more pronounced (27.5 vs. 13.9), indicating that vitamin D supplementation as adjuvant therapy can significantly enhance clinical efficacy (39). Another randomized placebo-controlled study confirmed that adding a single dose of 50,000 IU vitamin D3 to conventional glucocorticoid therapy significantly improved hearing at 2000 Hz and 4,000 Hz (*p* = 0.004 and *p* = 0.001), with particularly notable benefits at speech-critical frequencies, without inducing abnormal blood calcium or phosphorus levels (40). However, not all studies support the efficacy of acute-phase supplementation. A prospective cohort study showed that although the proportion of vitamin D deficiency in SSNHL patients was significantly higher than in controls (38.8% vs. 10.0%), vitamin D supplementation immediately after diagnosis for 3 months did not significantly improve hearing recovery rates (75.7% vs. 68.2%) or symptoms (14).

It is essential to interpret these trial results with caution. The two positive RCTs differ substantially in dosing (1500–2000 IU/day vs. 50,000 IU/week), treatment duration (10 days vs. 3 weeks), baseline 25(OH)D definitions of deficiency (<30 ng/mL vs. <20 ng/mL), and concurrent conventional therapies, making direct comparison and pooled interpretation inappropriate. Furthermore, both positive RCTs enrolled exclusively vitamin D-deficient patients, limiting the generalizability of findings to unselected SSNHL populations. The absence of benefit in the prospective cohort—which used lower-dose supplementation (800 IU/day D2 + 250 IU/day D3) over 3 months—may reflect insufficient dosing, delayed initiation, or the inherently different biological context of cohort versus randomized designs. Collectively, current evidence is insufficient to recommend vitamin D supplementation as a standard adjunct for all SSNHL patients; its role, if any, may be limited to selected subgroups with confirmed deficiency. This suggests that vitamin D deficiency may more significantly function as a modifiable risk factor affecting onset and recurrence rather than as a decisive target for acute-phase intervention, emphasizing the preventive significance of maintaining adequate long-term vitamin D levels in high-risk populations.

### 5.2 Synergistic treatment mechanism with glucocorticoids

Available evidence suggests that vitamin D status may significantly influence the responsiveness to glucocorticoid treatment in SSNHL. A cross-sectional study found that after glucocorticoid treatment, all vitamin D-sufficient patients achieved hearing recovery, while 87.5% of vitamin D-deficient patients had incomplete recovery, indicating a close relationship between vitamin D levels and glucocorticoid efficacy (2). This phenomenon may stem from vitamin D’s regulatory effects on glucocorticoid signaling pathways. Although related mechanisms have not been directly validated in otology, basic research suggests that vitamin D can enhance glucocorticoid sensitivity by upregulating glucocorticoid receptor expression, inhibiting pro-inflammatory factor release, and enhancing anti-inflammatory effects (36). Furthermore, inflammation plays an important role in SSNHL pathogenesis, with significantly elevated peripheral blood neutrophils, leukocytes, and immune-inflammatory indicators, with neutrophil levels positively correlated with the degree of hearing loss (41). Vitamin D’s immunomodulatory function may form synergistic anti-inflammatory effects with glucocorticoids by inhibiting the NF-κB pathway and reducing the production of IL-6 and TNF-*α*, collectively improving the inner ear microenvironment (36). It is noteworthy that although current mainstream clinical guidelines strongly recommend glucocorticoids as initial treatment for SSNHL (within 2 weeks of symptom onset) (42, 43), vitamin D status assessment or supplementation recommendations have not been incorporated, reflecting that this synergistic strategy still requires more high-quality evidence.

### 5.3 Bioavailability comparison of different administration routes

Current research on vitamin D administration routes in SSNHL treatment is limited, but clinical trials have employed different approaches. One randomized controlled study used oral vitamin D3 (single dose of 50,000 IU) combined with conventional treatment and observed significant increases in serum vitamin D levels with noticeable hearing improvement (40); another used daily doses of 1,500–2000 IU orally for 10 days, also achieving good efficacy (39). These results indicate that the oral route can effectively elevate *in vivo* vitamin D levels and produce clinical benefits. In contrast, no studies have specifically evaluated intravenous, intramuscular, or local administration (such as intratympanic) routes in SSNHL. Notably, glucocorticoids themselves have multiple administration methods, including systemic oral, intravenous, and intratympanic injection. Studies have shown that intratympanic glucocorticoid injection initiated within 18 days of symptom onset can significantly improve hearing recovery (*p* < 0.001) (44), while single-dose versus divided-dose oral glucocorticoids showed no significant difference in efficacy at 3 months (45). However, none of these studies involved comparisons of different vitamin D administration routes. Given that vitamin D is a fat-soluble vitamin whose oral absorption depends on bile and intestinal function, its bioavailability may be affected by individual metabolic status. Future research should systematically compare different administration routes (e.g., high-dose intermittent oral vs. low-dose continuous oral vs. non-oral routes) for their efficiency in elevating serum 25-hydroxyvitamin D concentrations, tissue distribution, and clinical outcomes to optimize supplementation strategies.

## 6 Controversies and limitations of current research

### 6.1 Causal inference challenges in observational studies

Most current evidence regarding the association between vitamin D and SSNHL derives from cross-sectional or case–control studies. Although these observational designs can reveal statistical correlations, establishing causal relationships is difficult. Multiple studies consistently found that serum 25-hydroxyvitamin D levels in SSNHL patients were significantly lower than in healthy controls (1–3, 18), with vitamin D deficiency more prevalent among patients (3), suggesting its possible involvement in disease onset. However, these associations must be interpreted with caution. Potential confounders include: (i) sun exposure and seasonal variation, as patients with acute illness may have reduced outdoor activity; (ii) systemic inflammatory burden, as elevated IL-6 and other cytokines in SSNHL may suppress hepatic 25-hydroxylation, resulting in lower 25(OH)D as a consequence rather than a cause of disease; (iii) lifestyle and dietary patterns, which simultaneously influence vitamin D status and cardiovascular risk; and (iv) comorbidities such as diabetes, hypertension, and obesity, which are independently associated with both vitamin D deficiency and hearing impairment. More importantly, large-scale two-sample Mendelian randomization analysis based on European populations showed no significant causal association between genetically predicted serum 25-hydroxyvitamin D levels and idiopathic SSNHL (IVW method: OR = 1.09, 95% CI = 0.81–1.48, *p* = 0.573), with no evidence of heterogeneity or horizontal pleiotropy (15). This null finding from Mendelian randomization—which is less susceptible to confounding and reverse causation than observational studies—substantially challenges the causal interpretation of observational associations. Consequently, vitamin D should be more cautiously framed as an associated candidate biomarker rather than a proven mechanistic driver of SSNHL. Future causal inference frameworks, including cross-ethnic Mendelian randomization studies using non-European GWAS data, are needed to clarify this relationship. Additionally, a prospective cohort study indicated that vitamin D deficiency is a modifiable risk factor for SSNHL onset and recurrence, especially with the strongest association in those under 30 years, but short-term vitamin D supplementation after diagnosis did not significantly improve hearing recovery rates (14), further highlighting the complexity of transitioning from association to causal inference.

### 6.2 Confounding effects of vitamin D receptor polymorphisms

The biological effects of vitamin D are highly dependent on VDR function, and the VDR gene has multiple single nucleotide polymorphisms (such as FokI, BsmI, ApaI, TaqI) that may affect receptor expression, ligand binding affinity, and downstream signal transduction efficiency, thereby interfering with the association between vitamin D and hearing outcomes. Although zebrafish models have confirmed that vdrb gene knockdown leads to abnormal inner ear development and reduced hair cells (46), suggesting a key role of the VDR pathway in the auditory system, direct evidence for VDR polymorphisms in human SSNHL research remains insufficient. It is important to clearly distinguish between: (a) theoretical molecular plausibility—well established through *in vitro* and animal models demonstrating that VDR variants alter transcriptional efficiency of vitamin D target genes; and (b) clinically validated evidence in SSNHL—currently absent, as no human studies have systematically evaluated VDR polymorphism effects on SSNHL susceptibility or hearing recovery. Existing literature mostly focuses on VDR polymorphism effects in other disease contexts, such as VDR BsmI polymorphism associated with rheumatoid arthritis susceptibility (47), FokI ff genotype associated with low vitamin D and high MMP-9 levels in coronary artery disease (48), and rs731236 genotype modifying vitamin D deficiency risk in knee osteoarthritis (49). In the SSNHL field, no high-quality studies have systematically evaluated the modulating effect of VDR polymorphisms on the relationship between vitamin D status and hearing prognosis. Notably, one study of elderly populations found no significant association between serum vitamin D levels and VDR TaqI polymorphisms (50), while another review pointed out the lack of direct evidence for vitamin D and SSNHL in the context of VDR polymorphisms (51). Furthermore, vitamin D levels themselves are influenced by genetic factors such as VDR gene polymorphisms, which may become potential confounding variables in explaining plasma 25(OH)D concentrations (52); if not controlled in research design, this may lead to biased effect estimates.

### 6.3 Uncertainty in optimal treatment dosage and duration

Although some randomized controlled trials have shown that vitamin D supplementation can improve hearing recovery rates and tinnitus symptoms in SSNHL patients (39, 40), there is no unified standard regarding optimal treatment dosage, administration frequency, or treatment duration. Existing intervention studies have employed widely varying doses: one study used 50,000 IU of vitamin D3 per week (40), another used 1,500–2000 IU daily (39), and yet another administered 4,000 IU daily (53), with all reporting positive outcomes but lacking head-to-head comparisons to determine optimal dosage. Additionally, treatment duration is inconsistent, ranging from 10 days to 3 months (39), with the necessity of long-term maintenance treatment yet to be established. Furthermore, the definition of “vitamin D deficiency” varies across studies (e.g., <12, <20, or <30 ng/mL), creating non-comparable patient populations; outcome measures differ in audiometric endpoints, frequency ranges assessed, and follow-up duration; and the concurrent use of glucocorticoids, *ginkgo biloba*, and other agents confounds the attribution of benefit to vitamin D specifically. Notably, a prospective cohort study found that short-term vitamin D supplementation after diagnosis did not significantly improve hearing recovery rates, suggesting that maintaining adequate long-term vitamin D levels may be more critical for preventing onset or recurrence (14), creating tension with acute-phase treatment strategies. No guidelines currently recommend specific vitamin D supplementation protocols for SSNHL patients, and the uncertainty in optimal dosage and duration limits standardized application in clinical practice.

## 7 Future research directions

### 7.1 Standardized design of prospective cohort studies

Current research on the association between vitamin D and SSNHL is mostly cross-sectional or retrospective in design, making it difficult to establish causal relationships. Although a prospective cohort study has indicated that vitamin D deficiency is a modifiable risk factor for SSNHL onset and recurrence, especially with the strongest association in those under 30 years (14), short-term vitamin D supplementation after diagnosis did not significantly improve hearing recovery rates, suggesting that intervention timing and duration may have decisive effects on efficacy. Therefore, there is an urgent need for large-scale, multicenter, long-term follow-up prospective cohort studies with unified inclusion criteria, vitamin D deficiency definitions (e.g., serum 25-hydroxyvitamin D < 20 ng/mL), hearing assessment methods (e.g., pure-tone audiometry frequency range and recovery criteria), and treatment regimens. Additionally, clear distinction should be made between idiopathic SSNHL and other secondary etiologies (such as acoustic neuroma) to reduce confounding bias (54). Through standardized design, the temporal relationship between dynamic changes in vitamin D levels and SSNHL onset, recurrence, and prognosis can be more accurately evaluated, providing high-quality evidence for causal inference.

### 7.2 Precision classification through vitamin D metabolomics

Metabolomics technology has provided new perspectives for revealing the potential biological mechanisms of SSNHL. Research has found that amino acid metabolism, lipid metabolism, purine and pyrimidine metabolism, and autophagy pathways are closely associated with acquired sensorineural hearing loss, with differentially expressed metabolites such as sphingosine potentially serving as diagnostic or prognostic biomarkers (55). Given that vitamin D is itself a fat-soluble hormone whose metabolic process involves multiple enzymatic steps including hepatic 25-hydroxylation and renal 1α-hydroxylation, with significant individual heterogeneity in metabolic efficiency, future research can integrate targeted and non-targeted metabolomics methods to systematically analyze the concentration profiles of vitamin D and its active metabolites (such as calcitriol) in SSNHL patient plasma and perilymph, combined with audiometric phenotypes (such as trough-shaped audiograms, high-frequency, or low-frequency hearing loss) for cluster classification. For example, studies have shown that serum 25(OH)D levels are linearly positively correlated with low-frequency and speech-frequency hearing loss but negatively correlated with high-frequency hearing loss, with specific threshold effects (e.g., 35.3–53.9 nmol/L) (56). The integration of vitamin D metabolomics within a broader precision medicine framework—encompassing nutrigenomics, pharmacogenomics, and individualized preventive strategies—represents a promising approach for identifying SSNHL patient subgroups most likely to benefit from targeted vitamin D interventions (57). Through metabolomics-driven precision classification, it is expected that SSNHL subgroups most sensitive to vitamin D intervention can be identified, achieving individualized treatment.

### 7.3 Multicenter validation of gene–environment interactions

The effects of vitamin D are influenced not only by environmental factors (such as sunlight, diet, and season) but also highly dependent on genetic background, especially VDR gene polymorphisms and metabolic enzyme gene variants. Although no studies directly investigating the association between VDR polymorphisms and SSNHL currently exist, whole-genome sequencing has revealed that the expression patterns of genes such as NOTCH1, APOE, and RELB are significantly associated with hearing recovery (58), suggesting that genetic factors play an important role in SSNHL prognosis. Meanwhile, while Mendelian randomization studies have not found a causal association between serum 25(OH)D levels and SSNHL (15), this conclusion is based on European population GWAS data and may be limited by sample representativeness or the complexity of gene–environment interactions. Notably, diabetes has been shown to have a significant interaction with 25(OH)D in low-frequency hearing loss (*p* = 0.041) (56), suggesting that chronic metabolic status may modulate the protective effects of vitamin D. Therefore, cross-ethnic, multicenter gene–environment interaction studies should be conducted, integrating whole-genome data, vitamin D metabolic indicators, lifestyle factors, and comorbidity information to build predictive models, clarifying how genetic susceptibility modulates the protective effects of vitamin D on inner ear function and providing a basis for early screening and intervention in high-risk populations.

## 8 Conclusion

Synthesizing current evidence, vitamin D likely exerts protective effects in the onset and progression of SSNHL through multiple synergistic biological pathways, including regulation of calcium metabolism, immunomodulation, vascular endothelial protection, and mitigation of oxidative stress—pathways that converge within cochlear pathophysiology through VDR-mediated transcriptional networks. Numerous clinical studies consistently demonstrate that serum 25-hydroxyvitamin D levels are significantly lower in SSNHL patients compared to healthy controls. Furthermore, vitamin D deficiency is associated with more severe hearing loss, poorer therapeutic response, and higher recurrence risk, suggesting that serum vitamin D concentration holds exploratory potential clinical value as a candidate biomarker for risk stratification and prognostic assessment in SSNHL.

Nevertheless, the causal relationship between vitamin D and SSNHL remains controversial. Mendelian randomization analyses based on European populations have failed to identify a causal link between genetically predicted vitamin D levels and idiopathic SSNHL, and this null causal finding must be accorded appropriate weight in interpreting the clinical relevance of observational associations. Existing epidemiological evidence may be confounded by unmeasured factors or reverse causation bias. Additionally, heterogeneity across studies regarding definitions of vitamin D deficiency, supplementation dosages, and treatment durations has led to inconsistent conclusions about intervention efficacy: while some randomized controlled trials report significant improvements in hearing recovery with vitamin D supplementation, others observe no clear benefit. These discrepancies, compounded by the small sample sizes of existing trials and the absence of head-to-head dosing comparisons, mean that current evidence does not support recommending vitamin D supplementation as a standard adjunctive therapy for unselected SSNHL patients. This evidence gap suggests that the clinical utility of vitamin D as a therapeutic target may lie primarily in personalized interventions for specific patient subgroups and long-term prevention strategies, rather than as a universally applicable acute-phase supplementation approach. Standardized application therefore requires further support from high-quality evidence.

In conclusion, vitamin D exhibits dual attributes in SSNHL as both a candidate biomarker and a potential adjunctive therapeutic consideration; however, current evidence remains insufficient to endorse its integration into routine clinical practice. Based on the totality of available data—including the null finding from Mendelian randomization—vitamin D is more appropriately framed as a promising adjunctive biomarker candidate than as a validated therapeutic target ready for routine clinical incorporation. Future research should prioritize large-scale prospective cohort studies and multicenter randomized controlled trials conducted under unified methodological standards. Integrating vitamin D metabolomics and analysis of vitamin D receptor (VDR) gene polymorphisms will be essential to identify SSNHL patient subgroups most likely to benefit from vitamin D intervention. Such efforts will facilitate the translation of research findings into precision medicine approaches, ultimately enhancing overall prevention, treatment outcomes, and prognosis quality for patients with SSNHL.

## References

[1] Zheng H Liu M Fu H Zhao L. Serum fat-soluble vitamin levels may be related to sudden sensorineural hearing loss. Acta Otolaryngol. (2023) 143:576–81. doi: 10.1080/00016489.2023.2220365, 37466376

[2] Ghazavi H Kargoshaie AA Jamshidi-Koohsari M. Investigation of vitamin D levels in patients with sudden sensory-neural hearing loss and its effect on treatment. Am J Otolaryngol. (2020) 41:102327. doi: 10.1016/j.amjoto.2019.102327, 31735446

[3] Zandi A Mehrad-Majd H Afzalzadeh MR. Association between serum vitamin D levels and risk of sudden sensorineural hearing loss: a cross-sectional study. Indian J Otolaryngol Head Neck Surg. (2023) 75:2345–52. doi: 10.1007/s12070-023-03917-9 37974694 PMC10646042

[4] Dwivedi S Singh V Sen A Yadav D Agrawal R Kishore S . Vitamin D in disease prevention and cure-part I: an update on molecular mechanism and significance on human health. Indian J Clin Biochem. (2025) 40:339–81. doi: 10.1007/s12291-024-01251-7, 40625600 PMC12229305

[5] Büki B Jünger H Zhang Y Wang Y Lundberg YW. The price of immune responses and the role of vitamin D in the inner ear. Otol Neurotol. (2019) 40:701–9. doi: 10.1097/MAO.0000000000002258, 31194714 PMC6578582

[6] Liang R Wang W Gao W Lu L Chen X Zhang Y . Calcitriol alleviates noise-induced hearing loss by regulating the ATF3/DUSP1 signalling pathway. Ecotoxicol Environ Saf. (2024) 284:116906. doi: 10.1016/j.ecoenv.2024.116906 39182283

[7] Bigman G. Deficiency in vitamin D is associated with bilateral hearing impairment and bilateral sensorineural hearing loss in older adults. Nutr Res. (2022) 105:1–10. doi: 10.1016/j.nutres.2022.05.008, 35779352

[8] Szeto B Valentini C Lalwani AK. Low vitamin D status is associated with hearing loss in the elderly: a cross-sectional study. Am J Clin Nutr. (2021) 113:456–66. doi: 10.1093/ajcn/nqaa310, 33247302

[9] Nocini R Henry BM Mattiuzzi C Lippi G. Serum vitamin D concentration is lower in patients with tinnitus: a meta-analysis of observational studies. Diagnostics (Basel). (2023) 13:1037. doi: 10.3390/diagnostics13061037, 36980345 PMC10047354

[10] Mehta CH Close MF Dornhoffer JR Liu YF Nguyen SA McRackan TR . Vitamin D deficiency, hypocalcemia, and hearing loss in children. Otol Neurotol. (2020) 41:940–7. doi: 10.1097/MAO.0000000000002676, 32658400

[11] Nowaczewska M Osiński S Marzec M Wiciński M Bilicka K Kaźmierczak W. The role of vitamin D in subjective tinnitus-a case-control study. PLoS One. (2021) 16:e0255482. doi: 10.1371/journal.pone.0255482, 34407088 PMC8372974

[12] Wu Y Lai Z Li A Han W Liu X Fan W. Association of low serum 25-hydroxyvitamin D levels with hearing loss severity in Meniere disease: a cross-sectional study. Front Neurol. (2025) 16:1638357. doi: 10.3389/fneur.2025.1638357, 40959501 PMC12434304

[13] Molnár A Mavrogeni P Mavrogenis A Maihoub S. Levels of 25-hydroxyvitamin D in individuals with primary subjective tinnitus and their associations with tinnitus occurrence and severity. Front Neurol. (2026) 16:1751366. doi: 10.3389/fneur.2025.1751366., 41602974 PMC12832500

[14] Hui Q Xiang T Qinglan X Shuowei Y Mengshi C Qin X . Vitamin D deficiency as a risk factor for onset and recurrence of sudden sensorineural hearing loss: a prospective cohort study with age-specific analysis. Food Sci Nutr. (2026) 14:e71383. doi: 10.1002/fsn3.71383, 41541701 PMC12803506

[15] Zhao Y Yu C Sun H Xie F Shen J Li X . Exploring the causal link between serum 25-hydroxyvitamin D concentrations and idiopathic sudden sensorineural hearing loss: insights gained from a mendelian randomization study involving two independent samples. PLoS One. (2025) 20:e0322898. doi: 10.1371/journal.pone.0322898, 40388403 PMC12087992

[16] Noh K Chow ECY Quach HP Groothuis GMM Tirona RG Pang KS. Significance of the vitamin D receptor on crosstalk with nuclear receptors and regulation of enzymes and transporters. AAPS J. (2022) 24:71. doi: 10.1208/s12248-022-00719-9, 35650371

[17] Bayraktar E Lopez-Pigozzi D Bortolozzi M. Calcium regulation of connexin hemichannels. Int J Mol Sci. (2024) 25:6594. doi: 10.3390/ijms25126594, 38928300 PMC11204158

[18] Karaer I Akalın Y. Low vitamin B12 level and vitamin D level adversely affect on cochlear health in women. Int J Vitam Nutr Res. (2020) 90:333–8. doi: 10.1024/0300-9831/a000616, 31623529

[19] Liu T He R Tian Y Chen W Liu X Zhu L . Cochlear immunology and its therapeutic revolution. Front Immunol. (2025) 16:1666224. doi: 10.3389/fimmu.2025.1666224, 41306961 PMC12644081

[20] Kim HB Lim SH Cho CG Choi HS. Influence of vitamin D deficiency on progression of experimental otitis Media in Rats. Endocrinol Metab (Seoul). (2018) 33:296–304. doi: 10.3803/EnM.2018.33.2.296, 29947185 PMC6021308

[21] Büki B Jünger H Lundberg YW. Vitamin D supplementation may improve symptoms in Meniere's disease. Med Hypotheses. (2018) 116:44–6. doi: 10.1016/j.mehy.2018.04.019, 29857909 PMC6023719

[22] Saleem N Robbins M Foster J Kelley B. Hearing loss in leucine-rich glioma-inactivated 1 encephalitis: Cochlear implantation considerations. Cureus. (2025) 17:e89296. doi: 10.7759/cureus.89296, 40904981 PMC12404648

[23] Umashankar A Prabhu P. Hearing loss and hypertension: a literature review. Indian J Otolaryngol Head Neck Surg. (2022) 74:532–40. doi: 10.1007/s12070-021-02378-2, 36032913 PMC9411486

[24] Weiss BG Bertlich M Bettag SA Desinger H Ihler F Canis M. Drug-induced Defibrinogenation as new treatment approach of acute hearing loss in an animal model for inner ear vascular impairment. Otol Neurotol. (2017) 38:648–54. doi: 10.1097/MAO.0000000000001400, 28369007

[25] Costantino VV Gil Lorenzo AF Bocanegra V Vallés PG. Molecular mechanisms of hypertensive nephropathy: renoprotective effect of losartan through Hsp70. Cells. (2021) 10:3146. doi: 10.3390/cells10113146, 34831368 PMC8619557

[26] Mahmoud AM Szczurek M Hassan C Masrur M Gangemi A Phillips SA. Vitamin D improves nitric oxide-dependent vasodilation in adipose tissue arterioles from bariatric surgery patients. Nutrients. (2019) 11:2521. doi: 10.3390/nu11102521, 31635396 PMC6835261

[27] Bertlich M Ihler F Weiss BG Freytag S Jakob M Strupp M . Fingolimod (FTY-720) is capable of reversing tumor necrosis factor induced decreases in cochlear blood flow. Otol Neurotol. (2017) 38:1213–6. doi: 10.1097/MAO.0000000000001510, 28742634

[28] Sharaf K Ihler F Bertlich M Reichel CA Berghaus A Canis M. Tumor necrosis factor-induced decrease of cochlear blood flow can be reversed by etanercept or JTE-013. Otol Neurotol. (2016) 37:e203–8. doi: 10.1097/MAO.0000000000001095, 27295443

[29] Zhang X Zhou K Tian K Zhu Q Liu W Liu Z . VDR regulates BNP promoting neurite growth and survival of cochlear spiral ganglion neurons through cGMP-PKG signaling pathway. Cells. (2022) 11:3746. doi: 10.3390/cells11233746, 36497006 PMC9739822

[30] Li W Zhou Y Yang H Shi X She W. Microcirculatory dysfunction and oxidative stress in sudden sensorineural hearing loss: insights from a case-control and experimental study. Med Sci Monit. (2026) 32:e950766. doi: 10.12659/MSM.950766, 41851951 PMC13011658

[31] Shin SA Lyu AR Jeong SH Kim TH Park MJ Park YH. Acoustic trauma modulates cochlear blood flow and vasoactive factors in a rodent model of noise-induced hearing loss. Int J Mol Sci. (2019) 20:5316. doi: 10.3390/ijms20215316, 31731459 PMC6862585

[32] Dong Y Liu D Hu Y Ma X. NaHS protects cochlear hair cells from gentamicin-induced ototoxicity by inhibiting the mitochondrial apoptosis pathway. PLoS One. (2015) 10:e0136051. doi: 10.1371/journal.pone.0136051, 26295804 PMC4546415

[33] Bener A Eliaçık M Cincik H Öztürk M DeFronzo RA Abdul-Ghani M. The impact of vitamin D deficiency on retinopathy and hearing loss among type 2 diabetic patients. Biomed Res Int. (2018) 2018:1–8. doi: 10.1155/2018/2714590, 30112372 PMC6077590

[34] Tang EKY Tieu EW Tuckey RC. Expression of human CYP27B1 in Escherichia coli and characterization in phospholipid vesicles. FEBS J. (2012) 279:3749–61. doi: 10.1111/j.1742-4658.2012.08736.x, 22862690

[35] Usategui-Martín R De Luis-Román DA Fernández-Gómez JM Ruiz-Mambrilla M Pérez-Castrillón JL. Vitamin D receptor (VDR) gene polymorphisms modify the response to vitamin D supplementation: a systematic review and meta-analysis. Nutrients. (2022) 14:360. doi: 10.3390/nu14020360, 35057541 PMC8780067

[36] Hamayal M Khurshied S Zahid MA eKhurshid N Shahid W Ali M . Exploring the significance of vitamin D levels as a biomarker in ear diseases: a narrative review. Cureus. (2024) 16:e54812. doi: 10.7759/cureus.54812 38529449 PMC10962011

[37] Okuda H Aoki M Ohashi T Ogawa B Shibata H Ueda N . Serum fibrinogen level and cytokine production as prognostic biomarkers for idiopathic sudden sensorineural hearing loss. Otol Neurotol. (2022) 43:e712–9. doi: 10.1097/MAO.0000000000003552, 35802892

[38] Al-Azzawi A Stapleton E. Blood tests as biomarkers for the diagnosis and prognosis of sudden sensorineural hearing loss in adults: a systematic review. J Laryngol Otol. (2023) 137:977–84. doi: 10.1017/S0022215123000282, 36794400

[39] Shen X Yang M Tian J Xie L Feng N Ma R. Clinical efficacy of vitamin D combined with conventional therapy for sudden sensorineural hearing loss in patients with vitamin D deficiency: a randomized controlled trial. Head Face Med. (2025) 21:68. doi: 10.1186/s13005-025-00545-2, 41053896 PMC12502239

[40] Tavakoli B Rabiei S. The impact of vitamin D supplementation on sudden sensorineural hearing loss in vitamin D deficient patients: a double-blind, placebo-controlled trial: a pilot study. Am J Otolaryngol. (2026) 47:104793. doi: 10.1016/j.amjoto.2026.104793, 41570758

[41] Akin V Yasan H Sivrice ME Kumbul YÇ. Examination of pan-immune-inflammation value and lymphocyte-monocyte ratio in sudden sensorineural hearing loss. Niger J Clin Pract. (2025) 28:243–7. doi: 10.4103/njcp.njcp_246_24, 40326908

[42] Chandrasekhar SS Tsai Do BS Schwartz SR Bontempo LJ Faucett EA Finestone SA . Clinical practice guideline: sudden hearing loss (update) executive summary. Otolaryngol Head Neck Surg. (2019) 161:195–210. doi: 10.1177/0194599819859883, 31369349

[43] Chandrasekhar SS Tsai Do BS Schwartz SR Bontempo LJ Faucett EA Finestone SA . Clinical practice guideline: sudden hearing loss (update). Otolaryngol Head Neck Surg. (2019) 161:S1. doi: 10.1177/0194599819859885 31369359

[44] Kennedy DJ Williams E Wolf L Golden J Lana A Wiefels M . Intratympanic steroid administration and predictors of recovery in sudden sensorineural hearing loss. PLoS One. (2025) 20:e0332809. doi: 10.1371/journal.pone.0332809, 41066339 PMC12510503

[45] Yu GH Choi YJ Jung HJ Lim YS Park SW Cho CG . A comparison of single-dose and multiple divided daily-dose oral steroids for sudden sensorineural hearing loss. Braz J Otorhinolaryngol. (2019) 85:733–8. doi: 10.1016/j.bjorl.2018.06.001, 30056032 PMC9443028

[46] Kwon HJ. Vitamin D receptor deficiency impairs inner ear development in zebrafish. Biochem Biophys Res Commun. (2016) 478:994–8. doi: 10.1016/j.bbrc.2016.08.070, 27526995

[47] Ahmad HM Zaki ZM Mohamed AS Ahmed AE. Genetic risk of rheumatoid arthritis: a case control study. Biochem Genet. (2024) 62:3624–41. doi: 10.1007/s10528-023-10648-7, 38160213 PMC11427610

[48] Moradi N Fadaei R Ahmadi R Mohammad MH Shahmohamadnejad S Tavakoli-Yaraki M . Role of serum MMP-9 levels and vitamin D receptor polymorphisms in the susceptibility to coronary artery disease: an association study in Iranian population. Gene. (2017) 628:295–300. doi: 10.1016/j.gene.2017.07.060, 28739397

[49] Arellano Pérez Vertti RD Arellano Ramírez DO González Galarza FF Prieto Hinojosa AI Méndez Hernández A Muñoz Flores MV . Association of vitamin D blood deficiency and the rs731236 polymorphism vitamin D receptor with primary knee osteoarthritis in subjects from Mexico. Clin Rheumatol. (2025) 44:1329–35. doi: 10.1007/s10067-025-07332-z, 39849258

[50] de Souza Freitas R Fratelli CF de Souza Silva CM de Lima LR Stival MM da Silva ICR . Association of vitamin D with the TaqI polymorphism of the VDR gene in older women attending the basic health unit of the Federal District, DF (Brazil). J Aging Res. (2020) 2020:7145193. doi: 10.1155/2020/7145193 33029399 PMC7532410

[51] Tamasauskiene L Golubickaite I Ugenskiene R Sjakste N Paramonova N Wu LS . Vitamin D receptor gene polymorphisms in atopy. Immunity Inflamm Dis. (2021) 9:1153–9. doi: 10.1002/iid3.487, 34343413 PMC8589349

[52] Kowalówka M Główka AK Karaźniewicz-Łada M Kosewski G. Clinical significance of analysis of vitamin D status in various diseases. Nutrients. (2020) 12:2788. doi: 10.3390/nu12092788, 32933052 PMC7551674

[53] Paprocki J Sutkowy P Piechocki J Woźniak A. Association between vitamin D supplements, oxidative stress biomarkers, and hyperbaric therapy in patients with sudden sensorineural hearing loss. Oxidative Med Cell Longev. (2021) 2021:8895323. doi: 10.1155/2021/8895323, 33777323 PMC7972839

[54] Hosokawa K Hosokawa S Takebayashi S Mineta H. Trough-shaped audiograms are common in patients with acoustic neuroma and sudden sensorineural hearing loss. Audiol Neurootol. (2018) 23:58–62. doi: 10.1159/000490233, 30021194

[55] Wang S Xie M Wu Z Wang S Tang Q Li C . Application of metabolomics to acquired hearing loss: advances and systematic review. Hear Res. (2025) 464:109302. doi: 10.1016/j.heares.2025.109302, 40532493

[56] Chen F Gao Y Wang Y Pan Z Chen Y Sheng H . Association of serum 25-hydroxyvitamins D_2_ and D_3_ with hearing loss in US adults: analysis from National Health and nutrition examination survey, 2015-2016. Front Nutr. (2024) 11:1390953. doi: 10.3389/fnut.2024.1390953, 39131738 PMC11310169

[57] Saini S Rathore A Suresh N Singh V. Vitamins a, B, C, D, E, and K as molecular modulators: integrative roles in precision medicine, nutrigenomics, therapy, and disease prevention. Indian J Precis Med Mol Med. (2025) 1:44–52. doi: 10.4103/IJPMMM.IJPMMM_10_25

[58] Yang A Liu S Yang X Guo Z Li J Li X . Uncovering novel prognostic factors of sudden sensorineural hearing loss by whole-genome sequencing of cell-free DNA. J Int Adv Otol. (2022) 18:459–64. doi: 10.5152/iao.2022.21493, 36349665 PMC9682783

